# *Syringa reticulata* potently inhibits the activity of SARS-CoV-2 3CL protease

**DOI:** 10.1016/j.bbrep.2023.101626

**Published:** 2023-12-26

**Authors:** Zhichao Hao, Yuan Liu, Wei Guan, Juan Pan, MengMeng Li, Jiatong Wu, Yan Liu, Haixue Kuang, Bingyou Yang

**Affiliations:** Key Laboratory of Chinese Materia Medica, Ministry of Education of Heilongjiang University of Chinese Medicine, No. 24 Haping Road, Xiangfang District, Harbin, 150040, PR China

**Keywords:** COVID-19, SARS-CoV-2, 3CL protease, *Syringa reticulata*, Traditional Chinese medicine

## Abstract

The ongoing coronavirus infectious disease (COVID-19) pandemic caused by severe acute respiratory syndrome coronavirus 2 (SARS-CoV-2) still urgently requires effective treatments. The 3C-like (3CL) protease of SARS-CoV-2 is a highly conserved cysteine protease that plays an important role in the viral life cycle and host inflammation, providing an ideal target for developing broad-spectrum antiviral drugs. Herein, we describe the discovery of a large number of herbs mainly produced in Heilongjiang Province, China, that exhibited different inhibitory activities against SARS-CoV-2 3CL protease. We confirmed that *Syringa reticulata*, which is used for clinical treatment of chronic bronchitis and asthma, is a specific and potent inhibitor of 3CL protease. A 70 % ethanol extract of *S. reticulata* dose-dependently inhibited the cleavage activity of 3CL protease in a fluorescence resonance energy transfer assay with an IC_50_ value of 0.0018 mg/mL, but had minimal effect in pseudovirus-based cell entry and luciferase-based RNA-dependent RNA polymerase assays. These results suggest that *S. reticulata* will be a potential leading candidate for COVID-19 treatment.

## Highlights

• 61 herbs produced in Heilongjiang province of China have diverse inhibitory activity against SARS-CoV-2 3CL protease.
• *Syringa reticulata* exhibits extraordinary inhibitory activity against 3CL protease compared to other herbs.
• *Syringa reticulata* specifically inhibits 3CL protease activity with minimal effect in viral entry and RdRp activity.

## 1 Introduction

The outbreak and subsequent pandemic of a novel coronavirus pneumonia (COVID-19) caused by severe acute respiratory syndrome coronavirus 2 (SARS-CoV-2) in 2019 caused unexpected impacts on public health and economic losses [1,2]. Until now, the number of SARS-CoV-2 infections reported by The World Health Organization (WHO) has exceeded 657 million, with 6.68 million deaths (https://covid19.who.int/). Various countries, including China, have urgently approved several vaccines and clinical drugs, including various traditional Chinese medicines (TCMs), for the prevention and treatment of COVID-19 [1,[3], [4], [5]]. However, because of the large number of mutant strains [6] and the bottleneck of new drug development, the development of safe, effective, and cost-efficient drugs is still urgently required [7].

SARS-CoV-2 is a single-stranded, positive-sense, enveloped RNA virus with a total genome of 30 kb, which is composed of at least 29 open reading frames that encode four structural proteins (spike, membrane, envelope, and nucleocapsid), 16 nonstructural proteins (NSP1–16), and several accessory proteins [8]. Papain-like protease (NSP3) and 3C-like protease (3CL protease, NSP5) have crucial proteolytic activities that cleave the polyprotein precursors to release the 16 nonstructural proteins to initiate virus replication [9,10]. 3CL protease is also called main protease owing to its responsibility for specifically recognizing 11 cleavage sites between NSP5 and NSP16, whereas papain-like protease recognizes three cleavage sites between NSP1 and NSP4 [11]. Moreover, 3CL protease plays an important role in the cytokine storm caused by SARS-CoV-2. Mehdi Moustaqil and colleagues demonstrated that the 3CL protease of SARS-CoV-2 specifically mediates cleavage of two inflammatory factors, TAB1 and NLPR12, enhancing the production of a multiple inflammatory cytokines and the immune response [12]. Crucially, there are no human proteases with similar cleavage activities to 3CL protease, making this as an ideal and specific target against which to develop broad-spectrum anti-viral drugs [13].

TCMs and formulae are widely used, especially in treating cases of mild symptoms, and exhibit considerable advantages by directly inhibiting SARS-CoV-2 replication and reducing the expression of inflammatory factors [14]. Lianhuaqingwen capsule, a Chinese patent medicine comprising 13 herbs, was approved for marketing to treat cold and influenza by the National Medical Products Administration of China in 2004 and has been demonstrated to significantly inhibit the replication of SARS-CoV-2 and reduce production of proinflammatory cytokines [5]. In addition, TCM has unique advantages such as rich resources, low costs, and minimal side effects and thus represents ideal drug libraries to develop leading compounds against SARS-CoV-2 [15].

*Syringa reticulata*, a member of the Oleaceae family, is widely used in the treatment of chronic bronchitis, cough, asthma, and hypertension [16,17]. *S. reticulata* contains numerous components such as iridoids, phenylpropanoids, flavonoids, phenylethanoid glycosides, and volatile oils that possess antibacterial, anti-inflammatory, and antioxidant activities [18,19]. In this study, we used the FRET-based 2019-nCoV Mpro/3CLpro Inhibitor Screening Kit to screen SARS-CoV-2 3CL protease inhibitory activity of 61 herbs mainly produced in Heilongjiang Province of China and identified *S. reticulata* to have a significant and specific inhibitory activity against 3CL protease compared with that in other traditional antiviral herbs, including *Scutellaria baicalensis*, *Forsythia suspensa*, and *Isatis tinctoria*. Finally, we provide data that suggest *S. reticulata* is potential candidate from which to develop broadly active 3CL protease inhibitors.

## 2 Materials and methods

### 2.1 Cell culture

Baby hamster kidney 21/hACE2 cells (BHK-21/hACE2) and human embryonic kidney 293T (HEK-293T) cells were obtained from Procell (Wuhan, China). Cells were maintained in Dulbecco's modified Eagle medium (DMEM, Gibco, Suzhou, China) supplemented with 10 % fetal bovine serum (FBS) (Gibco, Suzhou, China), 1 % streptomycin, and 1 % penicillin (Gibco, Suzhou, China) at 37 °C.

### 2.2 Herbs and herbal extracts

Sixty-one dried herbs were collected from Heilongjiang Province of China and identified by Professor Rui-Feng Fan of the Heilongjiang University of Chinese Medicine. These herbs were ground into powder and sieved through a 40-mesh sieve. Powder samples (5 g) were extracted using ultrasonication with 100 mL of 70 % ethanol for 60 min. After filtering and completely removing the solvent, the residue was freeze-dried overnight. Dried herbal residue was completely dissolved in water to obtain 100 mg/mL stock solution.

### 2.3 Inhibitory activity of 3CL protease assay

The 2019-nCoV Mpro/3CLpro Inhibitor Screening Kit (Beyotime, Shanghai, China) was used to confirm the inhibitory activity of 3CL protease according to the manufacturer's instructions. Briefly, 5 μL of water or serial dilutions of extracted herbs were preincubated with 93 μL of diluted SARS-CoV-2 3CL protease (10 μg/mL) for 15 min at room temperature. Then 2 μL of fluorescence substrate was added to initiate the reaction. The relative fluorescence units (RFU) with an excitation wavelength of 390 nm and emission wavelength of 490 nm were recorded after 5 min incubation using a multimode plate reader (PerkinElmer, Waltham, USA). Results were performed in two independent experiments. Inhibition (%) = (RFU100 % protease activity − RFUSamples)/(RFU100 % protease activity − RFUBlank) × 100 %.

### 2.4 Pseudovirus mediated entry assay

A codon-optimized spike protein gene from strain Wuhan-Hu-1 (GeneBank: NC_045512.2) was purchased from Sinobiological (spike-pCMV3, VG40589-UT); pCAG-dR8.9 and pCDH-NanoLuciferase were kept in our laboratory. SARS-CoV-2 spike pseudovirus system was produced by our laboratory. Briefly, HEK-293T cells were transfected with spike-pCMV3, pCAG-dR8.9, and pCDH-NanoLuciferase at a 1:1:2 ratio using Polyjet (SignaGen, Maryland, USA) following the manufacturer's instructions. After 6 h, the complete medium was discarded and changed with DMEM containing 2 % FBS. SARS-CoV-2 pseudovirus containing culture supernatants was harvested 48 h post transfection and 0.45-μm filtered and stored at −80 °C until use. The neutralization assay was performed using 5 mg/mL *S. reticulata* extract incubated with pseudovirus for 1 h at 37 °C together with the virus control and cell control. The mixture was then incubated with BHK-21/hACE2 cells for 24 h. Luminescence was measured using Nano-Glo Luciferase Assay System (Promega, Madison, USA) according to the manufacturer's instructions.

### 2.5 RdRP activity assay

SARS-CoV-2 RdRp activity assay was performed as previously described [20]. Briefly, pCMV3-RdRp-Flag (SinoBiological, Beijing, China), pcDNA3.1-NSP7, pcDNA3.1-NSP8, and pcDNA3.1-reporter (synthesized by Comate, Changchun, China) were co-transfected into HEK-293T cells at a 10:30:30:1 ratio, whereas pcDNA3.1-reporter was transfected alone as negative control. Then *S. reticulata* extract (5 mg/mL) was introduced to the medium after 6 h transfection; the luminescence was measured using Nano-Glo Luciferase Assay System (Promega, **Madison, USA) after another 24 h incubation.**

### 2.6 Statistical analysis

Data analysis was performed using GraphPad Prism 9 software. Differences among the different groups were determined based on one-way analysis of variance (ANOVA) followed by Tukey's multiple comparisons. p < 0.05 was considered significant.

## 3 Results

### 3.1 Primary screening of sixty-one herbs against the SARS-CoV-2 3CLprotease

**Fig. 1:**
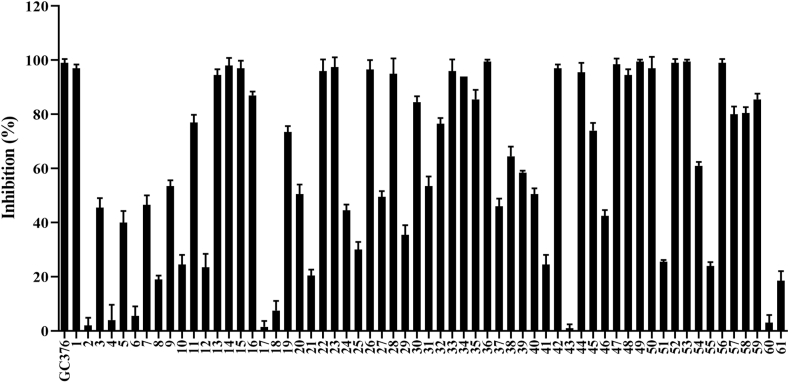
Screening of 61 traditional Chinese herbs against SARS-CoV-2 3CL protease using FRET assay. 70 % ethanol extracts (5 mg/ml) of these herbs were pre-incubated with SARS-CoV-2 3CL protease for 15 min at room temperature, and then 2 μL of fluorescence substrate was added to initiate the reaction. After 5 min reaction, RFU were recorded using a multimode plate reader. The results are mean ± SD deviation of two repeats.

Viral protease is an attractive antiviral drug target for RNA viruses, including SARS-CoV-2. In response to the COVID-19 pandemic, significant effort has been made to evaluate the possibility of various protease inhibitor drugs for the clinical treatment of this disease. To address this need, 61 herbs produced in Heilongjiang Province (Table 1) were screened using the Beyotime Mpro/3CLpro Inhibitor Screening Kit (Fig. 1). The extracts of 28 of these herbs (1, 11, 13–16, 22–23, 26, 28, 30, 32–36, 42, 44, 47–50, 52–53, and 56–59) showed >75 % inhibition against 3CL protease at 5 mg/mL, which included several traditional antiviral herbs (*S. baicalensis*, *Tripterygium wilfordii*, and *F. suspensa*). Surprisingly, *I. tinctoria*, which is traditionally used in treatment with influenza virus, showed minimal effect on 3CL protease. In addition, the extracts of 10 herbs showed medium levels of inhibitory activity ranging from 50 % to 75 % (9, 19–20, 27, 31, 38–40, 45, and 54), and those of 23 herbs exhibited weak activities of <50 % inhibition (2–8, 10, 12, 17–18, 21, 24–25, 29, 37, 41, 43, 46, 51, 55, and 60–61).

**Table 1:** List of herb names tested against SARS-CoV-2 3CL protease (A-Z).

Number	Herb Name	Number	Herb Name
1	*Actaea cimicifuga* L.	32	*Paeoniae Radix Alba*
2	*Adenophora stricta Miq.*	33	*Paeoniae Radix Rubra*
3	*Alisma plantago-aquatica L.*	34	*Panax ginseng*
4	*Allium macrostemon Bunge*	35	*Paris verticillata*
5	*Anemarrhena asphodeloides*	36	*Phellodendron amurense*
6	*Arisaema amurense Maxim.*	37	*Phragmites australis*
7	*Aster tataricus* L. *f.*	38	*Physalis Calyx Seu Fructus*
8	*Astragalus membranaceus*	39	*Polygala tenuifolia Willd.*
9	*Atractylodes japonica*	40	*Polygonatum odoratum*
10	*Atractylodes lancea*	41	*Polygonatum sibiricum*
11	*Bupleurum chinense DC.*	42	*Polygonum aviculare* L.
12	*Cichorium intybus* L.	43	*Polygonum perfoliatum* L.
13	*Cirsium arvense*	44	*Potentilla discolor Bge.*
14	*Cirsium spicatum*	45	*Pulsatilla chinensis*
15	*Cistanche deserticola*	46	*Radix Saposhnikoviae*
16	*Clematis chinensis*	47	*Rhaponticum uniflorum*
17	*Coix chinensis Tod.*	48	*Rheum palmatum* L.
18	*Coix lacryma-jobi Linn.*	49	*Rhododendron dauricum* L.
19	*Conioselinum smithii*	50	*Sanguisorba officinalis* L.
20	*Cuscuta chinensis Lam.*	51	*Saposhnikovia divaricata*
21	*Dictamnus dasycarpus*	52	*Schisandra chinensis*
22	*Eleutherococcus senticosus*	53	*Scutellaria baicalensis*
23	*Forsythia suspensa*	54	*Silybum marianum*
24	*Fritillaria ussuriensis*	55	*Stellera chamaejasme* L.
25	*Gentiana cruciata* L.	56	*Syringa reticulata*
26	*Geum aleppicum*	57	*Taraxacum mongolicum*
27	*Glycyrrhiza uralensis*	58	*Tripterygium wilfordii*
28	*Hyoscyamus niger*	59	*Tussilago farfara*
29	*Isatis tinctoria*	60	*Typha angustifolia* L.
30	*Juncus effusus* L.	61	*White Ginseng*
31	*Neoalsomitra clavigera*

### 3.2 Identification of *S. reticulata* as a potent inhibitor against SARS-CoV-2 3CL protease

**Fig. 2:**
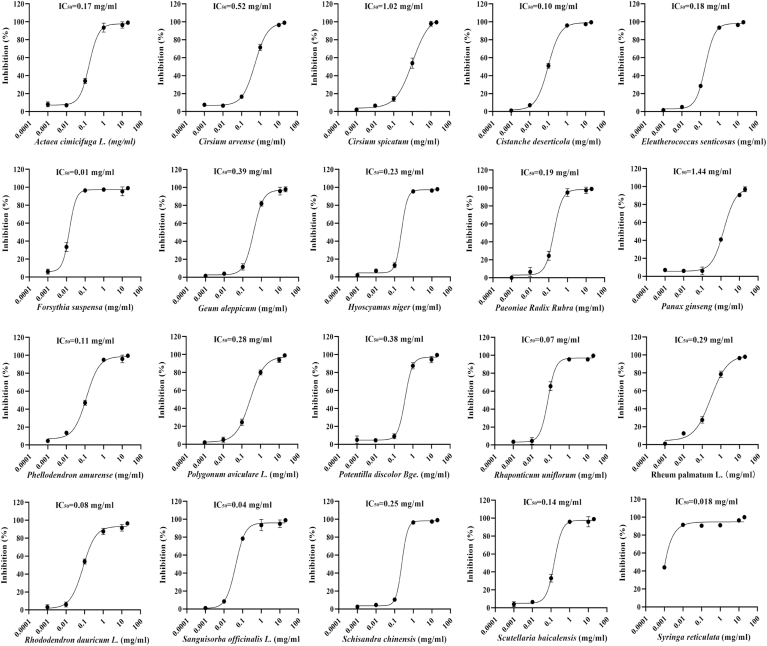
Dose-response relationships of 20 selected herbs against 3CL protease. 3CL protease were pre-incubated with different herbs at indicated concentration followed by 15 min incubation at room temperature, then 2 μL of fluorescence substrate was added to initiate the reaction. After 5 min reaction, RFU were recorded using a multimode plate reader, inhibitions were calculated as describe in Fig. 1 and expressed as mean ± SD of at two independent experiments.

To confirm the superior inhibitory activity of these herbs, we then further conducted dose-response profiling for 20 from these 28 herbs (inhibition >90 %) to test the inhibitory effect against 3CL protease (Fig. 2). *Panax ginseng* is an immune-enhancing agent that confers immunity against SARS-CoV-2 infection. However, in our secondary screening, *Panax ginseng* showed poorer inhibition of SARS-CoV-2 3CL protease with IC_50_ of 1.44 mg/mL. Notably, these 20 herbs have similar activity to inhibit 3CL protease activity at initial screening, but the *S. reticulata* led to 50 % inhibition of SARS-CoV-2 3CL protease activity at 0.0018 mg/mL, indicating significant activity against 3CL protease. Otherwise, *F. suspensa* and *S. baicalensis*, two key components of Tanreqing Injection, which relieves symptoms of fever, cough, and other discomfort caused by SARS-CoV-2 infection, also displayed medium and weak activity with IC_50_ values of 0.01 and 0.14 mg/mL compared to *S. reticulata*, respectively.

### 3.3 The *S. reticulata* is a specific SARS-CoV-2 3CL protease inhibitor

**Fig. 3:**
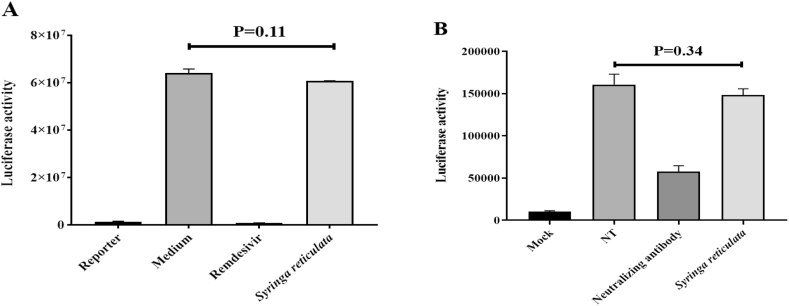
*Syringa reticulata* as specific 3CL protease inhibitor with minimum effects on RdRp activity and virus entry. (A) HEK-293T cells were co-transfected with pCMV3-RdRp-Flag, pcDNA3.1-NSP7, pcDNA3.1-NSP8 and pcDNA3.1-reporter at a 10:30:30:1 ratio or pcDNA3.1-reporter alone. 6 h post-transfection, 5 mg/ml of *Syringa reticulata* was introduced to the medium, Remdesivir as positive control. After 24 h incubation, the luminescence from each group was measured. (B) SARS-CoV-2 spike pseudotyped lentivirus were pre-incubated with 5 mg/ml of *Syringa reticulata* or DMEM (NT) for 1 h at 37 °C, neutralizing antibody as a positive control, the mixture of different groups was incubated with BHK-21/hACE2 cells for another 24 h, then the luminescence was determined using a multimode plate reader. The results are mean ± SD deviation of two repeats.

Spike-ACE2 interaction mediates virus entry and RdRp activity, which have a crucial role in the viral life cycle and are therefore important drug development targets. To elucidate whether *S. reticulata* extract could inhibit RdRp activity or block virus enter the target cell, we first used a cell-based assay to determine the inhibitory activity of *S. reticulata* extract on SARS-CoV-2 RdRp. As shown in Fig. 3A, when the Gaussia luciferase (Gluc) reporter-expressing cells were transfected with RdRp, the activity of Gluc dramatically increased compared with that of cells expressing the reporter alone, but *S. reticulata* extract had minimal inhibitory effect on RdRp activity. To demonstrate if *S. reticulata* extract could prevent infection by blocking SARS-CoV-2 cell entry, a pseudovirus-based neutralization assay was performed (Fig. 3B). We incubated pseudovirus with 5 mg/mL of *S. reticulata* extract or vehicle control (DMEM) and then infected human ACE2 over-expressing BHK-21 cells; however, we observed no apparent difference in cells treated with *S. reticulata* compared with that of vehicle control 24 h post-infection, (Fig. 3B). Collectively, we identified *S. reticulata* extract as a specific and potent drug targeting SARS-CoV-2 3CL protease but which had minimal effect on virus entry or RdRp activity.

## 4 Discussion

SARS-CoV-2 is an RNA virus with a large genome, and consequently has a relatively high mutation frequency accumulated during continuous transmission; this is especially relevant in the spike protein, where mutations cause escape from preexisting immunity for survival after wild-type strain-based vaccine injection [21]. The Pfizer/BioNTech and Moderna (mRNA-1273) prototype vaccines showed minimal effect on neutralizing the Beta and Omicron variants, and therefore potential leading drugs are urgently needed to target viral proteins that have indispensable and conserved roles [22]. Coronavirus 3CL protease, also known as main protease, is highly conserved in structure and has similar substrate specificities in all known coronavirus species because of the proteolytic processing of the replicase polyproteins [11]. 3CL protease shares >80 % homology within the Coronavirus genus, and the greatest degree of sequence conservation exists in and around the enzyme active site, which strongly supports the possibility of developing broadly active-site inhibitors that target SARS-CoV-2 3CL protease. For instance, Paxlovid, which consists of nirmatrelvir (SARS-CoV-2 3CL protease inhibitor) plus ritonavir (increases serum levels of nirmatrelvir), was highly effective in reducing the risk of severe COVID-19 or mortality and was approved for use by the National Medical Products Administration of China [23]. Another example is GC376, a dipeptide-based protease inhibitor widely used in feline-infectious peritonitis, could effectively inhibit the 3CL protease from both SARS-CoV and SARS-CoV-2 in the nanomolar range [24]. These all indicate that 3CL protease is a viable drug target because of its essential role in the viral life cycle.

In this study, 61 traditional Chinese herbs obtained from Heilongjiang Province of China were screened using a FRET-based commercial 3CL protease inhibitor screening assay. We found 28 herbs with >90 % inhibitory activity in our initial screening, including *Geum aleppicum*, *Schisandra chinensis*, *S. baicalensis*, *Cirsium spicatum*, *F. suspensa*, *Sanguisorba officinalis* L., and others. Among these herbs, the *S. baicalensis* extract dose-dependently inhibited the replication of SARS-CoV-2 by targeting 3CL protease and was shown to contain baicalein as an effective component [25]. In addition, Qinhai Ma reported that Phillyrin (KD-1), a representative ingredient of *F. suspensa*, has antiviral activity and decreased production of proinflammatory cytokines induced by SARS-CoV-2 [26]. Unfortunately, several traditional antiviral herbs, including *I. tinctoria* and *Glycyrrhiza uralensis*, displayed minimum activity against SARS-CoV-2 3CL protease with <50 % inhibition. Surprisingly, we identified *S. reticulata*, which is mainly used for clinical treatment of chronic bronchitis and asthma, as a potent inhibitory herb against SARS-CoV-2 3CL protease with an IC_50_ value of 0.0018 mg/mL. Currently, the use of S. reticulata for COVID-19 pneumonia treatment has not been reported, although several articles reported that the early stage of SARS CoV-2 infection is often accompanied with bronchitis and bronchopneumonia [27], two obvious symptoms that are the main therapeutic targets of *S. reticulata*, thereby suggesting that treatment with *S. reticulata* extract could reduce virus replication to relieve symptoms of bronchitis and bronchopneumonia caused by SARS-CoV-2 infection. Our previous study isolated a series of iridoid compounds from *S. reticulata* and confirmed their anti-inflammatory activities in vitro [16,19]. Lee-Huang and others reported that iridoid could inhibit the replications of VHSV, HIV, and HBV [[28], [29], [30]], suggesting that iridoid extracted from *S. reticulata* is a leading candidate for targeting 3CL protease.

Spike-mediated virus entry and RdRp play crucial roles in SARS-CoV-2 infection and genome replication and transcription and are the two additional attractive drug targets for SARS-CoV-2 [31,32]. Binding of the SARS-CoV-2 spike protein to the host cell surface receptor ACE2 or coreceptor is an essential step during the beginning of the viral life cycle. Multiple drugs targeting viral entry into host cells have been developed, including camostat-mesilate-T, chloroquine hydrochloride, abidol, and soluble rhACE2 as well as neutralizing antibodies [33]. After entry into the cells, SARS-CoV-2 employs a multisubunit replication and transcription complex (RTC), which is assembled by multiple NSPs cleaved by papain-like protease and 3CL protease from polyproteins, to accomplish the replication and transcription of the viral genome [34]. The RTC core component NSP12, which is the catalytic subunit and is also called RdRp, needs two other accessory factors, NSP7 and NSP8, to increase NSP12 binding to the template to catalyze the synthesis of a new viral RNA chain. Until now, many nucleotide and nucleoside analogs, including remdesivir, favipiravir, and ribavirin have been reported to disrupt viral replication or induce RNA mutation to prevent generation of progeny virus [35]. Our results show that although *S. reticulata* was the most effective 3CL protease inhibitor, this did not block SARS-CoV-2 spike-mediated pseudovirus entry nor exhibit any inhibitory activity against RdRp, indicating that *S. reticulata* was a specific and potent 3CL protease inhibitor against SARS-CoV-2.

In conclusion, we identified the 70 % ethanol extract of *S. reticulata* as an effective and specific inhibitory treatment that targeted SARS-CoV-2 3CL protease but did not affect virus entry or RdRp activity. This study highlights a potential opportunity to develop an effective leading candidate against existing and mutant SARS-CoV-2 variants.

## CRediT authorship contribution statement

**Zhichao Hao:** Conceptualization, Data curation, Writing – original draft. **Yuan Liu:** Methodology. **Wei Guan:** Writing – review & editing. **Juan Pan:** Writing – review & editing. **MengMeng Li:** Data curation. **Jiatong Wu:** Validation. **Yan Liu:** Writing – review & editing. **Haixue Kuang:** Investigation. **Bingyou Yang:** Project administration, Funding acquisition.

## Declaration of competing interest

The authors report no conflicts of interest. The authors alone are responsible for the content and writing of the paper.

## References

[1] Hu B., Guo H., Zhou P., Shi Z.L. (2021). Characteristics of sars-cov-2 and covid-19. Nat. Rev. Microbiol..

[2] To K.K., Sridhar S., Chiu K.H., Hung D.L., Li X., Hung I.F., Tam A.R., Chung T.W., Chan J.F., Zhang A.J., Cheng V.C., Yuen K.Y. (2021). Lessons learned 1 year after sars-cov-2 emergence leading to covid-19 pandemic. Emerg. Microb. Infect..

[3] Chung H., He S., Nasreen S., Sundaram M.E., Buchan S.A., Wilson S.E., Chen B., Calzavara A., Fell D.B., Austin P.C., Wilson K., Schwartz K.L., Brown K.A., Gubbay J.B., Basta N.E., Mahmud S.M., Righolt C.H., Svenson L.W., MacDonald S.E., Janjua N.Z., Tadrous M., Kwong J.C., Canadian Immunization Research Network Provincial Collaborative Network I (2021). Effectiveness of bnt162b2 and mrna-1273 covid-19 vaccines against symptomatic sars-cov-2 infection and severe covid-19 outcomes in ontario, Canada: test negative design study. BMJ.

[4] Najjar-Debbiny R., Gronich N., Weber G., Khoury J., Amar M., Stein N., Goldstein L.H., Saliba W. (2022). Effectiveness of paxlovid in reducing severe covid-19 and mortality in high risk patients. Clin. Infect. Dis..

[5] Runfeng L., Yunlong H., Jicheng H., Weiqi P., Qinhai M., Yongxia S., Chufang L., Jin Z., Zhenhua J., Haiming J., Kui Z., Shuxiang H., Jun D., Xiaobo L., Xiaotao H., Lin W., Nanshan Z., Zifeng Y. (2020). Lianhuaqingwen exerts anti-viral and anti-inflammatory activity against novel coronavirus (sars-cov-2). Pharmacol. Res..

[6] Zhao Y., Ni W., Liang S., Dong L., Xiang M., Cai Z., Niu D., Zhang Q., Wang D., Zheng Y., Zhang Z., Zhou D., Guo W., Pan Y., Wu X., Yang Y., Jing Z., Jiang Y., Chen Y., Yan H., Zhou Y., Xu K., Lan K. (2023). Vaccination with s(pan), an antigen guided by sars-cov-2 s protein evolution, protects against challenge with viral variants in mice. Sci. Transl. Med..

[7] Muralidar S., Ambi S.V., Sekaran S., Krishnan U.M. (2020). The emergence of covid-19 as a global pandemic: understanding the epidemiology, immune response and potential therapeutic targets of sars-cov-2. Biochimie.

[8] V'Kovski P., Kratzel A., Steiner S., Stalder H., Thiel V. (2021). Coronavirus biology and replication: implications for sars-cov-2. Nat. Rev. Microbiol..

[9] Motyan J.A., Mahdi M., Hoffka G., Tozser J. (2022). Potential resistance of sars-cov-2 main protease (mpro) against protease inhibitors: lessons learned from hiv-1 protease. Int. J. Mol. Sci..

[10] Ullrich S., Nitsche C. (2022). Sars-cov-2 papain-like protease: structure, function and inhibition. Chembiochem.

[11] Roe M.K., Junod N.A., Young A.R., Beachboard D.C., Stobart C.C. (2021). Targeting novel structural and functional features of coronavirus protease nsp 5 (3cl(pro), m(pro)) in the age of covid-19. J. Gen. Virol..

[12] Moustaqil M., Ollivier E., Chiu H.P., Van Tol S., Rudolffi-Soto P., Stevens C., Bhumkar A., Hunter D.J.B., Freiberg A.N., Jacques D., Lee B., Sierecki E., Gambin Y. (2021). Sars-cov-2 proteases plpro and 3clpro cleave irf3 and critical modulators of inflammatory pathways (nlrp12 and tab1): implications for disease presentation across species. Emerg. Microb. Infect..

[13] Zhang Y., Gao H., Hu X., Wang Q., Zhong F., Zhou X., Lin C., Yang Y., Wei J., Du W., Huang H., Zhou H., He W., Zhang H., Zhang Y., McCormick P.J., Fu J., Wang D., Fu Y., Lu X., Zhang T., Duan J., Qin B., Jiang H., Luo J., Zhang Y., Chen Q., Luo Q., Cheng L., Zhang Z., Zhang J., Li J. (2022). Structure-based discovery and structural basis of a novel broad-spectrum natural product against the main protease of coronavirus. J. Virol..

[14] An X., Zhang Y., Duan L., Jin D., Zhao S., Zhou R., Duan Y., Lian F., Tong X. (2021). The direct evidence and mechanism of traditional Chinese medicine treatment of covid-19. Biomed. Pharmacother..

[15] Liu M., Gao Y., Yuan Y., Yang K., Shi S., Zhang J., Tian J. (2020). Efficacy and safety of integrated traditional Chinese and western medicine for corona virus disease 2019 (covid-19): a systematic review and meta-analysis. Pharmacol. Res..

[16] Guo S., Liu Y., Sun Y.P., Pan J., Guan W., Li X.M., Wang S.Y., Algradi A.M., Yang B.Y., Kuang H.X. (2022). Four new secoiridoids from the stem barks of syringa reticulata (bl.) hara. Nat. Prod. Res..

[17] Machida K., Kaneko A., Hosogai T., Kakuda R., Yaoita Y., Kikuchi M. (2002). Studies on the constituents of syringa species. X. Five new iridoid glycosides from the leaves of syringa reticulata (blume) hara. Chem. Pharm. Bull. (Tokyo).

[18] Jin C., Jin M., Li R., Diao S., Sun J., Ma Y.J., Zhou W., Li G. (2020). Isolation of a new natural kingiside aglucone derivative and other anti-inflammatory constituents from syringa reticulata. Nat. Prod. Res..

[19] Liu Y., Zhu H.C., Guo S., Liu P., Zou H.D., Naseem A., Pan J., Guan W., Kuang H.X., Yang B.Y. (2022). Six new secoiridoid glycosides from the stem barks of syringa reticulata (bl.) hara. Fitoterapia.

[20] Zhao J., Guo S., Yi D., Li Q., Ma L., Zhang Y., Wang J., Li X., Guo F., Lin R., Liang C., Liu Z., Cen S. (2021). A cell-based assay to discover inhibitors of sars-cov-2 rna dependent rna polymerase. Antivir. Res..

[21] Saxena S.K., Kumar S., Ansari S., Paweska J.T., Maurya V.K., Tripathi A.K., Abdel-Moneim A.S. (2022). Characterization of the novel sars-cov-2 omicron (b.1.1.529) variant of concern and its global perspective. J. Med. Virol..

[22] Garcia-Beltran W.F., Lam E.C., St Denis K., Nitido A.D., Garcia Z.H., Hauser B.M., Feldman J., Pavlovic M.N., Gregory D.J., Poznansky M.C., Sigal A., Schmidt A.G., Iafrate A.J., Naranbhai V., Balazs A.B. (2021). Multiple sars-cov-2 variants escape neutralization by vaccine-induced humoral immunity. Cell.

[23] Mahase E. (2021). Covid-19: pfizer's paxlovid is 89% effective in patients at risk of serious illness, company reports. BMJ.

[24] Fu L., Ye F., Feng Y., Yu F., Wang Q., Wu Y., Zhao C., Sun H., Huang B., Niu P., Song H., Shi Y., Li X., Tan W., Qi J., Gao G.F. (2020). Both boceprevir and gc376 efficaciously inhibit sars-cov-2 by targeting its main protease. Nat. Commun..

[25] Liu H., Ye F., Sun Q., Liang H., Li C., Li S., Lu R., Huang B., Tan W., Lai L. (2021). Scutellaria baicalensis extract and baicalein inhibit replication of sars-cov-2 and its 3c-like protease in vitro. J. Enzym. Inhib. Med. Chem..

[26] Ma Q., Li R., Pan W., Huang W., Liu B., Xie Y., Wang Z., Li C., Jiang H., Huang J., Shi Y., Dai J., Zheng K., Li X., Hui M., Fu L., Yang Z. (2020). Phillyrin (kd-1) exerts anti-viral and anti-inflammatory activities against novel coronavirus (sars-cov-2) and human coronavirus 229e (hcov-229e) by suppressing the nuclear factor kappa b (nf-kappab) signaling pathway. Phytomedicine.

[27] Berezowska S., Lefort K., Ioannidou K., Ndiaye D.R., Maison D., Petrovas C., Rotman S., Piazzon N., Milowich D., Sala N., Tsai C.Y., Multone E., Bochud P.Y., Oddo M., Bisig B., de Leval L. (2021). Postmortem cardiopulmonary pathology in patients with covid-19 infection: single-center report of 12 autopsies from lausanne, Switzerland. Diagnostics.

[28] Lee-Huang S., Huang P.L., Zhang D., Lee J.W., Bao J., Sun Y., Chang Y.T., Zhang J., Huang P.L. (2007). Discovery of small-molecule hiv-1 fusion and integrase inhibitors oleuropein and hydroxytyrosol: Part i. Fusion [corrected] inhibition. Biochem. Biophys. Res. Commun..

[29] Micol V., Caturla N., Perez-Fons L., Mas V., Perez L., Estepa A. (2005). The olive leaf extract exhibits antiviral activity against viral haemorrhagic septicaemia rhabdovirus (vhsv). Antivir. Res..

[30] Zhao G., Yin Z., Dong J. (2009). Antiviral efficacy against hepatitis b virus replication of oleuropein isolated from jasminum officinale l. Var. Grandiflorum. J. Ethnopharmacol..

[31] Jackson C.B., Farzan M., Chen B., Choe H. (2022). Mechanisms of sars-cov-2 entry into cells. Nat. Rev. Mol. Cell Biol..

[32] Yin W., Mao C., Luan X., Shen D.D., Shen Q., Su H., Wang X., Zhou F., Zhao W., Gao M., Chang S., Xie Y.C., Tian G., Jiang H.W., Tao S.C., Shen J., Jiang Y., Jiang H., Xu Y., Zhang S., Zhang Y., Xu H.E. (2020). Structural basis for inhibition of the rna-dependent rna polymerase from sars-cov-2 by remdesivir. Science.

[33] Colson P., Rolain J.M., Raoult D. (2020). Chloroquine for the 2019 novel coronavirus sars-cov-2. Int. J. Antimicrob. Agents.

[34] Hillen H.S., Kokic G., Farnung L., Dienemann C., Tegunov D., Cramer P. (2020). Structure of replicating sars-cov-2 polymerase. Nature.

[35] Simonis A., Theobald S.J., Fatkenheuer G., Rybniker J., Malin J.J. (2021). A comparative analysis of remdesivir and other repurposed antivirals against sars-cov-2. EMBO Mol. Med..

